# The transcriptome profile of RPE cells by the fullerenol against hydrogen peroxide stress

**DOI:** 10.3389/fmed.2022.996280

**Published:** 2022-09-14

**Authors:** Xiaojun Wu, Fuwen Yao, Jing-Ying Xu, Jiao Chen, Ying Lu, Wei Li, Jing Deng, Lisha Mou, Qingling Zhang, Zuihui Pu

**Affiliations:** ^1^Department of Pathology, School of Basic Medical Sciences, Southern Medical University, Guangzhou, China; ^2^Department of Pathology, Guangdong Provincial People's Hospital, Guangdong Academy of Medical Sciences, Guangzhou, China; ^3^Department of Ophthalmology, Shenzhen Nanshan People's Hospital and the 6th Affiliated Hospital of Shenzhen University Health Science Center, Shenzhen, China; ^4^Department of Hepatopancreatobiliary Surgery, Institute of Translational Medicine, Shenzhen University Health Science Center, Shenzhen University School of Medicine, First Affiliated Hospital of Shenzhen University, Shenzhen Second People's Hospital, Shenzhen, China; ^5^Department of Pathology and Pathophysiology School of Medicine, Tongji University, China; ^6^Department of Biochemistry, College of Science, Northeastern University, Boston, MA, United States; ^7^Imaging Department, Institute of Translational Medicine, Shenzhen University Health Science Center, Shenzhen University School of Medicine, First Affiliated Hospital of Shenzhen University, Shenzhen Second People's Hospital, Shenzhen, China

**Keywords:** fullerenol, nanomaterial, RNA sequencing, oxidative stress, senescence, RPE, AMD

## Abstract

Age-related macular degeneration (AMD) causes central vision impairment with increased incidence. In the pathogenesis of AMD, reactive oxygen species (ROS) are associated with RPE cell apoptosis. H_2_O_2_ is an oxidative toxicant and is used to establish the AMD *in vitro* model. However, the mechanisms of ROS in H_2_O_2_-induced AMD are still unclear. Fullerenol, a promising antioxidant of nanomaterials, protects RPE cells from ROS attack. In addition to working as a scavenger, little is known about the antioxidant mechanism of fullerenol in RPE cells. In this study, transcriptome sequencing was performed to examine the global changes in mRNA transcripts induced by H_2_O_2_ in human ARPE-19 cells. Moreover, we comprehensively investigated the protective effects of fullerenol against H_2_O_2_-induced oxidative injury by RNA sequencing. Gene Ontology enrichment analysis showed that those pathways related to the release of positive regulation of DNA-templated transcription and negative regulation of apoptotic process were affected. Finally, we found that 12 hub genes were related to the oxidative-protection function of fullerenol. In summary, H_2_O_2_ affected these hub genes and signaling pathways to regulate the senescence of RPE cells. Moreover, fullerenol is a potent nanomaterial that protects the RPE and would be a promising approach for AMD prevention.

## Introduction

Aged-related macular degeneration (AMD) causes severe vision damage and loss by affecting the macular region of the retina (1). In Caucasians, for people aged over 70 (including 70), the overall early AMD prevalence is 13.2% (2). AMD is usually associated with the destruction of photoreceptors, abnormalities in the retinal pigment epithelium (RPE), and degeneration of the choriocapillaris (3, 4). The key contributor to AMD pathogenesis is oxidative damage-induced RPE senescence (5, 6). Oxidative stress can result in permanent cell senescence and overproduction of reactive oxygen species (ROS) (7). Previous studies have shown that reactive oxygen species contribute significantly to AMD. Accumulated ROS drives DNA damage, and the permanent DNA damage response induces the state of senescence of RPE cells (8, 9). Therefore, AMD can be prevented and delayed by avoiding oxidative damage to RPE cells.

Hydrogen peroxide (H_2_O_2_)-induced RPE damage is a method for establishing the AMD *in vitro* model (10–12). A previous study showed that a low concentration of H_2_O_2_ caused RPE senescence (13), and a high concentration caused cell death in a dose-dependent manner. Antioxidants, including melatonin (14), quercetin (15), farrerol (10), and kaempferol, can protect RPE cells from H_2_O_2_-induced apoptosis (16). In addition, H_2_O_2_ was reported to trigger necrosis in the RPE (17). Further investigation is needed to study H_2_O_2_-induced RPE cell senescence.

Fullerenol (C60[OH]n), derived from fullerene C60, shows great antioxidative potential in pharmaceutics and medical treatment of oxidative stress-related diseases (18). The good water solubility of fullerenol makes it useful in pharmaceutics and medical treatment of oxidative stress-related diseases (19, 20). It can remove free radicals such as superoxide anion radicals, hydroxyl radicals, lipid peroxyl radicals, and nitrous oxide radicals, making it effective in anti-aging, antioxidant stress, anti-inflammation, and anti-apoptosis (21, 22). In addition, ROS can bind to the electron-deficient position of fullerenol. As a result, fullerenol has the ability to reduce oxidative stress in cells (23). The cytotoxicity of fullerenol was proven to be low (24). Therefore, these advantages make fullerenol nanoparticles significant and promising in oxidative damage-induced disease research and treatment. Nanoparticle fullerenol was also proven to protect the RPE from oxidatively induced senescence by activating the SIRT1 pathway (25). However, the comprehensive transcriptional profile of fullerenol nanoparticles on senescent RPE is still unclear.

We comprehensively profiled the gene expression of ARPE-19 cells treated with H_2_O_2_ and/or fullerenol for the first time. We also identified 12 hub genes that were rescued by fullerenol treatment in H_2_O_2_-induced ARPE-19 senescent cells. These genes showed promise as therapeutic targets for AMD. In summary, our results provide evidence for a deeper investigation into the function of fullerenol nanoantioxidants in AMD treatment.

## Materials and methods

### Reagents

Fullerenol was synthesized by Jing-Ying Xu with previous published methods (26). The Senescence-Associated β-Galactosidase (SA-β-galactosidase) Staining Kit was from Cell Signaling Technology, Beverly, MA, USA. The PrimeScript TM RT Reagent Kit was from Takara, Dalian, China. TRIzolTM Reagent was purchased from Thermo Fisher Scientific (Waltham, MA, USA). DMEM/F12 1:1 (1X), FBS, P/S, and trypsin–EDTA were from GIBCO (Carlsbad, CA, USA), and the CCK-8 assay was performed with a cell counting kit by Yeasen Biotech (Jiangsu, China). 3% (w/w) H_2_O_2_ was purchased from Sigma Aldrich (St. Louis, MO, USA).

### Cell viability and *in situ* staining for SA-β-galactosidase activity

ARPE-19 cells were grown in 1:1 (1X) DMEM/F12 with 10% (v/v) FBS and 1% (v/v) P/S. Cells were passaged with 0.25% (v/v) trypsin/0.2% EDTA every 3–4 days. ARPE-19 cells were plated in a 96-well plate and treated at 80% confluence. Then, 3% (w/w) H_2_O_2_ was used to make a medium with the intended H_2_O_2_ concentration. H_2_O_2_ solution was freshly diluted each time. For the H_2_O_2_ exposure, the medium used for the cells was changed to DMEM/F12 with the desired concentration of H_2_O_2_. Before establishing the cell senescence model, the concentration of H_2_O_2_ best used for stimulating ARPE-19 cells was explored. H_2_O_2_ (0 μM, 50 μM, 100 μM, 150 μM, 200 μM, and 400 μM) diluted with the cell culture medium was tested to find the most suitable concentrations. For the fullerenol exposure, after 2 h of exposure to H_2_O_2_, 5ug/mL fullerenol was added and further incubated for 22 h before analysis. Cell viability was analyzed by CCK-8 assay following manufacturers instructions. Experiments were repeated at least 3 times. In the SA-β-galactosidase staining assay and RNA sequencing, the treatment of the H_2_O_2_ exposure group and fullerenol treatment group followed previous studies (25).

### RNA sequencing

The RNA-Seq Samples Consisted of Three Groups, Represented by the Control Group, the H_2_O_2_ Group, and the Fullerenol Group. For Each Group, Three Biological Replicates Were Considered, for a Total of 9 Samples. CDNA Libraries From These Samples Were Sequenced and Analyzed According to the Protocols for RNA-Seq (Novogene Company, Beijing, China). The Gene Expression Distribution of Each Sample That Passed Quality Control Was Used for Further Analysis (Supplementary Figures S1A–D).

### Identification of differentially expressed genes (DEGs)

DESeq2 R Pakage Is Used for Differential Analysis of Comparative High-Throughput Sequencing Assays, Based on Gene Count Data. Shrinkage Estimation for Dispersions and Fold Changes Are Used to Improve Stability and Interpretability of Estimates. DEGs of the Three Groups in Our Study Were Analyzed by the DESeq2 R Package (1.16.1) With Fold Change > 1.5 and *P* < 0.05. The Overlay of DEGs Was Performed by Evenn (Http://www.Ehbio.com/Test/Venn/#/).

### Gene ontology (GO) enrichment analysis

DAVID is a popular bioinformatics resource system including a web server and web service for functional annotation and enrichment analyses of gene lists. It consists of a comprehensive knowledge base and a set of functional analysis tools. We examined the DEGs by GO enrichment analysis of DAVID (https://david.ncifcrf.gov/) with a significance threshold of *P* < 0.05. Biological processes enriched by DEGs were obtained for further analysis.

### Functional network analysis of the hub genes

The potential roles and related genes of the genes were analyzed by GeneMANIA (27, 28). GeneMANIA identifies other genes that are associated with the input genes. GeneMANIA used very large datasets of functional association data including protein and genetic interactions, pathways, co-expression, co-localization, and similarity of the protein domain (27, 28).

Ingenuity Pathway Analysis software (IPA, QIAGEN) was used to analyze the potential regulatory networks and related diseases of the hub genes. Ingenuity Pathway Analysis (IPA) can interpret the biological changes, altered canonical pathways, upstream transcriptional regulators, and gene networks, which contains large knowledge of gene functions and interaction networks based on published literature collected by the Ingenuity Pathway Analysis software.

### Statistical analysis

The statistical analyses were conducted in R 4.0.5. The student's *t*-test was used for statistical analysis. *P* < 0.05 was considered statistically significant. The heatmap plots were computed and visualized using the R package pheatmap.

## Results

### H_2_O_2_-induced senescence in ARPE-19 cells

**Figure 1:**
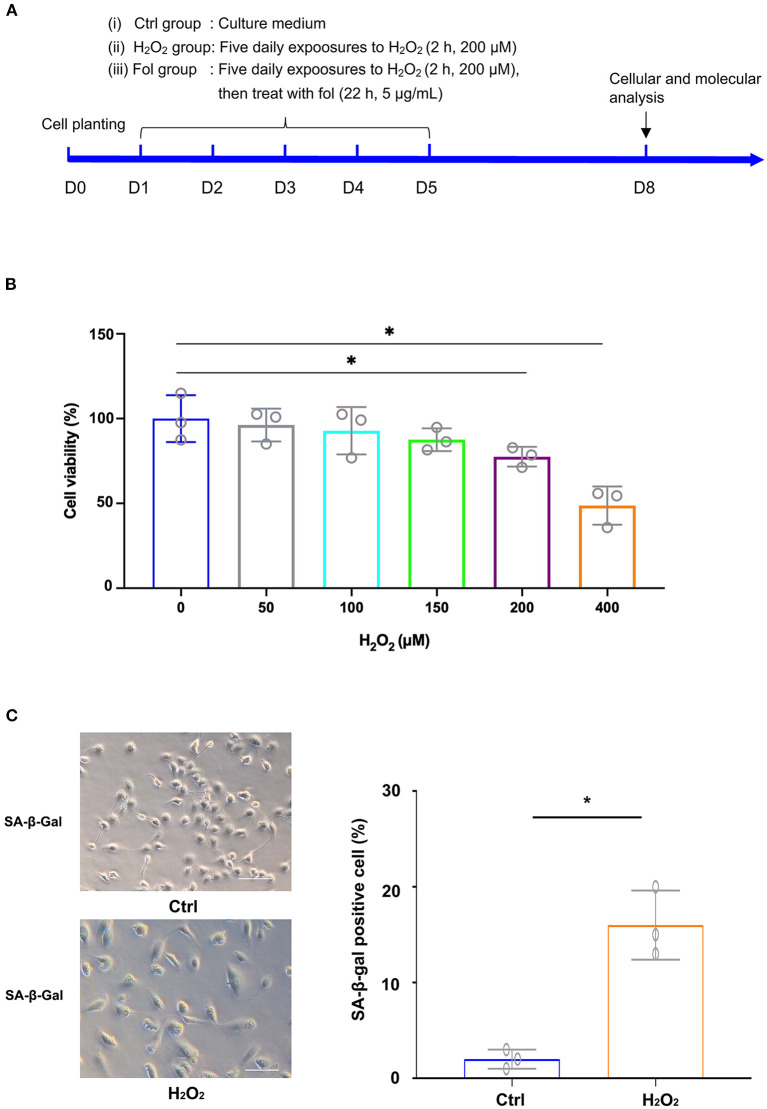
H_2_O_2_-Induced Senescence in ARPE-19 Cells. **(A)** Experimental design and reference time frame. **(B)** ARPE-19 cells were treated with H_2_O_2_ (50 μM, 100 μM, 150 μM, 200 μM, 400 μM) for 2 h daily. Cell viability was analyzed with the CCK8 method. **(C)** ARPE-19 cells in the control group and H_2_O_2_ group of ARPE-19 cells were stained with senescence-associated β-galactosidase (SA-β-Gal). Fol, fullerenol; *p < 0.05.

A cell senescence model and fullerenol treatment model were first established by exposing ARPE-19 cells to H_2_O_2_, as illustrated in Figure 1A. The cell cultures were exposed to H_2_O_2_ at various concentrations. As detected by the CCK-8 assay, statistical significance was found between the control and the H_2_O_2_-treated groups at 200 μM and 400 μM, respectively (Figure 1B). Thus, H_2_O_2_ at a 200 μM concentration was used in the following experiments. The results suggested that ARPE-19 cells treated with 200 μM H_2_O_2_ for 2 hours for five consecutive days were able to establish a senescence model, as confirmed by senescence-associated β-galactosidase staining (SA-β-galactosidase) (Figure 1C). In the control group, few cells were positive with SA-β-galactosidase staining (2%) (Figure 1C). In the H_2_O_2_ group, the ratio of cells that were positive with SA-β-galactosidase staining increased to 16% (Figure 1C).

### Analysis of differentially expressed mRNAs by RNA sequencing in H_2_O_2_-treated ARPE-19 cells

**Figure 2:**
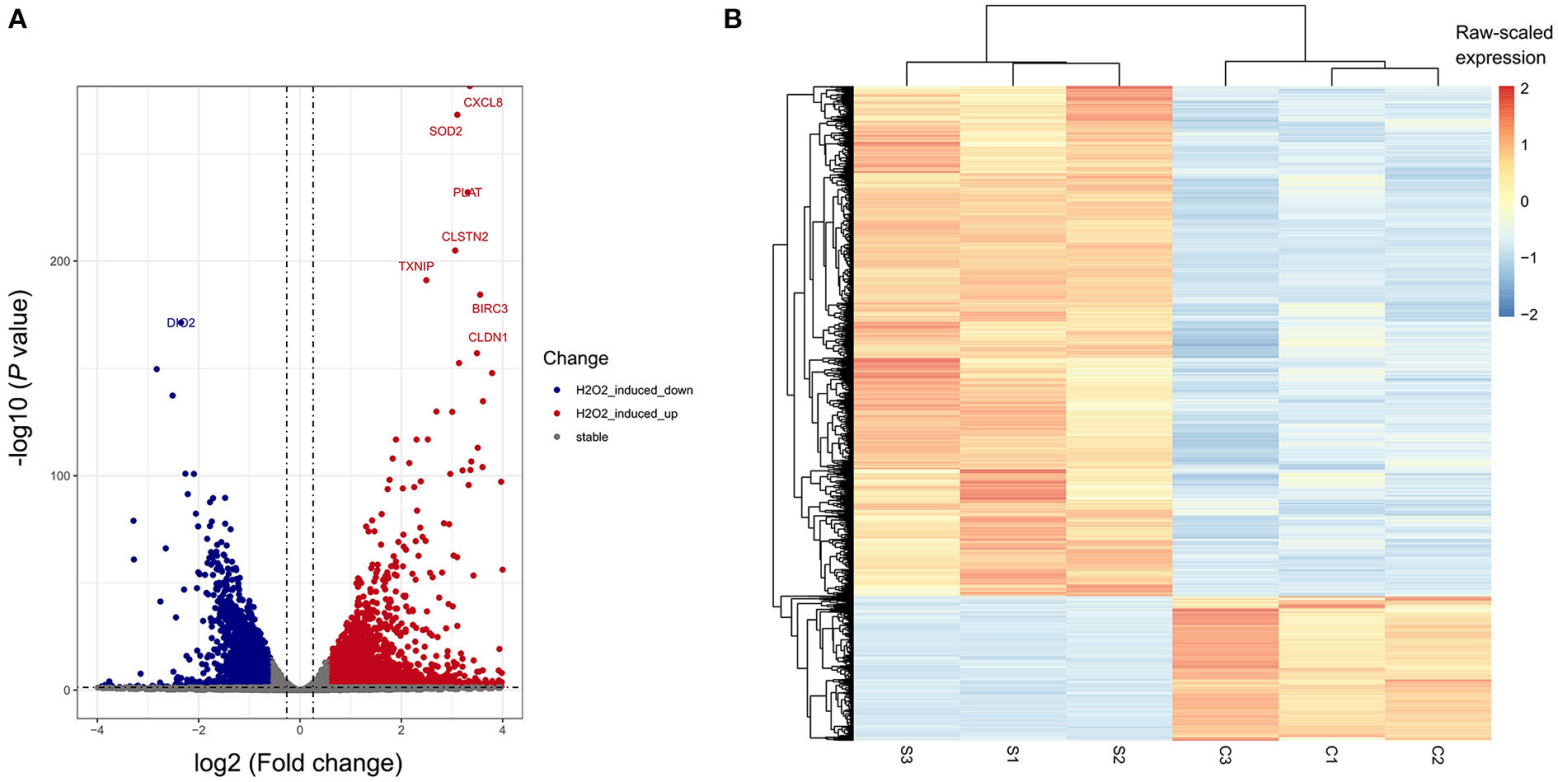
Differentially-expressed genes (DEGs) of H_2_O_2_-treated ARPE-19 cells. **(A)** Volcano plot of DEGs of RPE cells induced by H_2_O_2_. **(B)** Heatmap plot of DEGs of RPE cells induced by H_2_O_2_. S, H_2_O_2_ group; C, Ctrl group.

To determine the transcriptome profile of H_2_O_2_-treated ARPE-19 cells, we performed RNA sequencing of the H_2_O_2_-treated group and the control group of ARPE-19 cells, with each group containing three biological replicates. We performed differentially expressed gene analysis by DESeq2. In total 2,926 and 825 were considered as significantly up-or down- regulated genes after H_2_O_2_ treatment, respectively, compared with the control sample, respectively (Figures 2A,B). Among them, *CXCL8, SOD2, PLAT, CLSTN2, TXNIP, BIRC3*, and *CLDN1* were the top up-regulated genes after H_2_O_2_ treatment, while *DIO*_2_ was the top down-regulated gene (Figure 2A).

### Fullerenol-induced dynamic gene expression changes in H_2_O_2_-treated ARPE-19 cells

**Figure 3:**
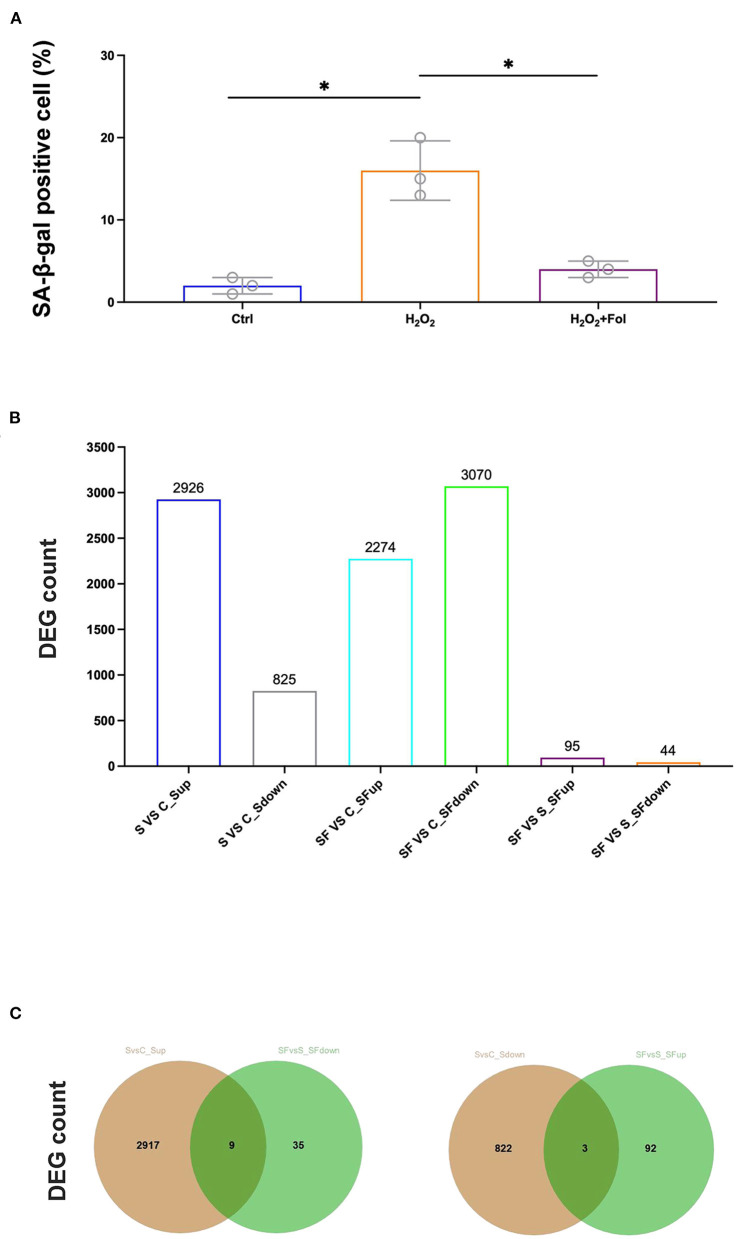
Hub genes and pathways affected by H_2_O_2_ and fullerenol. **(A)** ARPE-19 cells in the control group, H_2_O_2_ group, and fullerenol group were stained with SA-β-Gal. **(B)** Counts of DEGs among all the groups. **(C)** The overlay of genes up-regulated after H_2_O_2_ treatment and down-regulated after fullerenol treatment is showed in the left panel, while genes down-regulated after H_2_O_2_ treatment and up-regulated after fullerenol treatment is showed in the right panel. Fol, fullerenol; S, H_2_O_2_ group; C, Ctrl group; SF, Fullerenol group. *p < 0.05.

Fullerenol was shown to protect the RPE from oxidatively induced senescence in a previous study (25), which was also confirmed in our study (Figure 3A). Therefore, we performed RNA-seq to analyze the cytoprotective effect of fullerenol. DESeq2 was used to analyze differentially expressed genes with fold change ≥ 1.5 and *P* < 0.05 (Figure 3B). As a result, 2,926 and 825 genes were up- or downregulated after H_2_O_2_ induction in ARPE-19 cells, respectively (Figures 3B,C). At the same time, 2,274 and 3,070 genes were up- or downregulated after H_2_O_2_ and fullerenol treatment, respectively, indicating the dynamic gene expression regulation induced by fullerenol. Among them, we found that 95 and 44 genes were up- or downregulated between fullerenol and H_2_O_2_ treatment, respectively (Figure 3B). These genes might play crucial roles in the biological processes induced by fullerenol.

**Figure 4:**
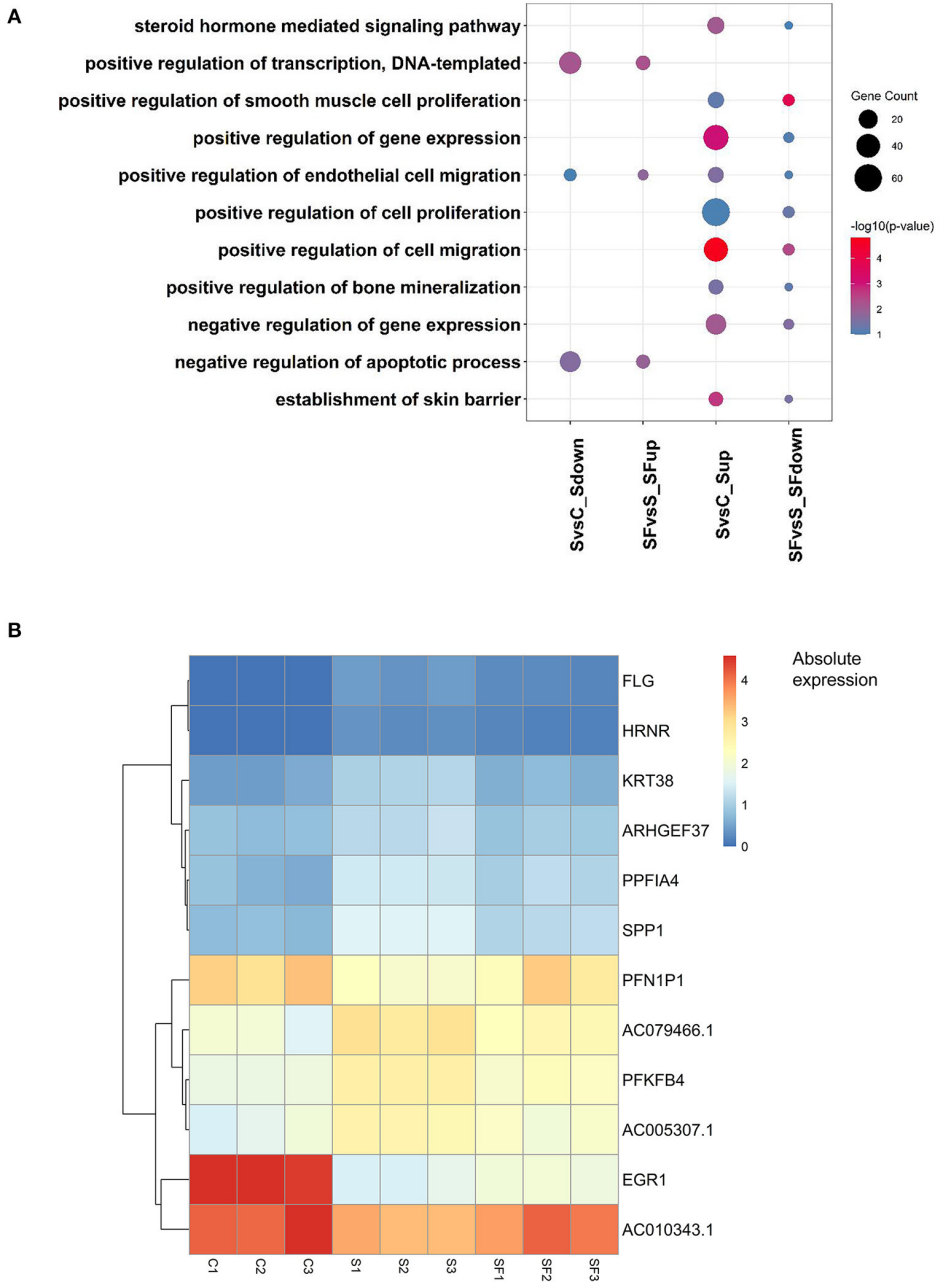
Functions and genes of genes rescued by fullerenol treatment. **(A)** Common GO terms of DEGs induced by H_2_O_2_ and fullerenol. **(B)** Heatmap plot of the expression of hub genes affected by H_2_O_2_ and fullerenol. S, H_2_O_2_ group; C, Ctrl group; SF, Fullerenol group.

Furthermore, after Gene Ontology enrichment by DEGs among the three groups, we found that 11 GO terms were enriched by the DEGs up-regulated after H_2_O_2_ but down-regulated after fullerenol treatment, or down-regulated after H_2_O_2_ but up-regulated after fullerenol treatment. Positive regulation of DNA-templated transcription, positive regulation of endothelial cell migration, and negative regulation of apoptotic process pathways were both enriched by DEGs down-regulated after H_2_O_2_ treatment and up-regulated after fullerenol treatment, indicating the potential roles of fullerenol (Figure 4A). At the same time, the steroid hormone-mediated signaling pathway, gene expression, cell proliferation, cell migration, and negative regulation of gene expression pathways were both enriched by DEGs up-regulated after H_2_O_2_ treatment and down-regulated after fullerenol treatment (Figure 4A). We further identified 9 genes (*ARHGEF37, KRT38, AC005307.1, FLG, HRNR, AC79466.1, PPFIA4, PFKFB4*, and *SPP1*) that were up-regulated after H_2_O_2_ treatment and down-regulated after fullerenol treatment (Figure 4B), and 3 genes (*EGR1, AC010343.1*, and *PFN1P1*) were down-regulated after H_2_O_2_ treatment and up-regulated after fullerenol treatment (Figure 4B). These pathways and genes were reversed by fullerenol in ARPE-19 cells.

### Interaction networks of the 12 hub genes

**Figure 5:**
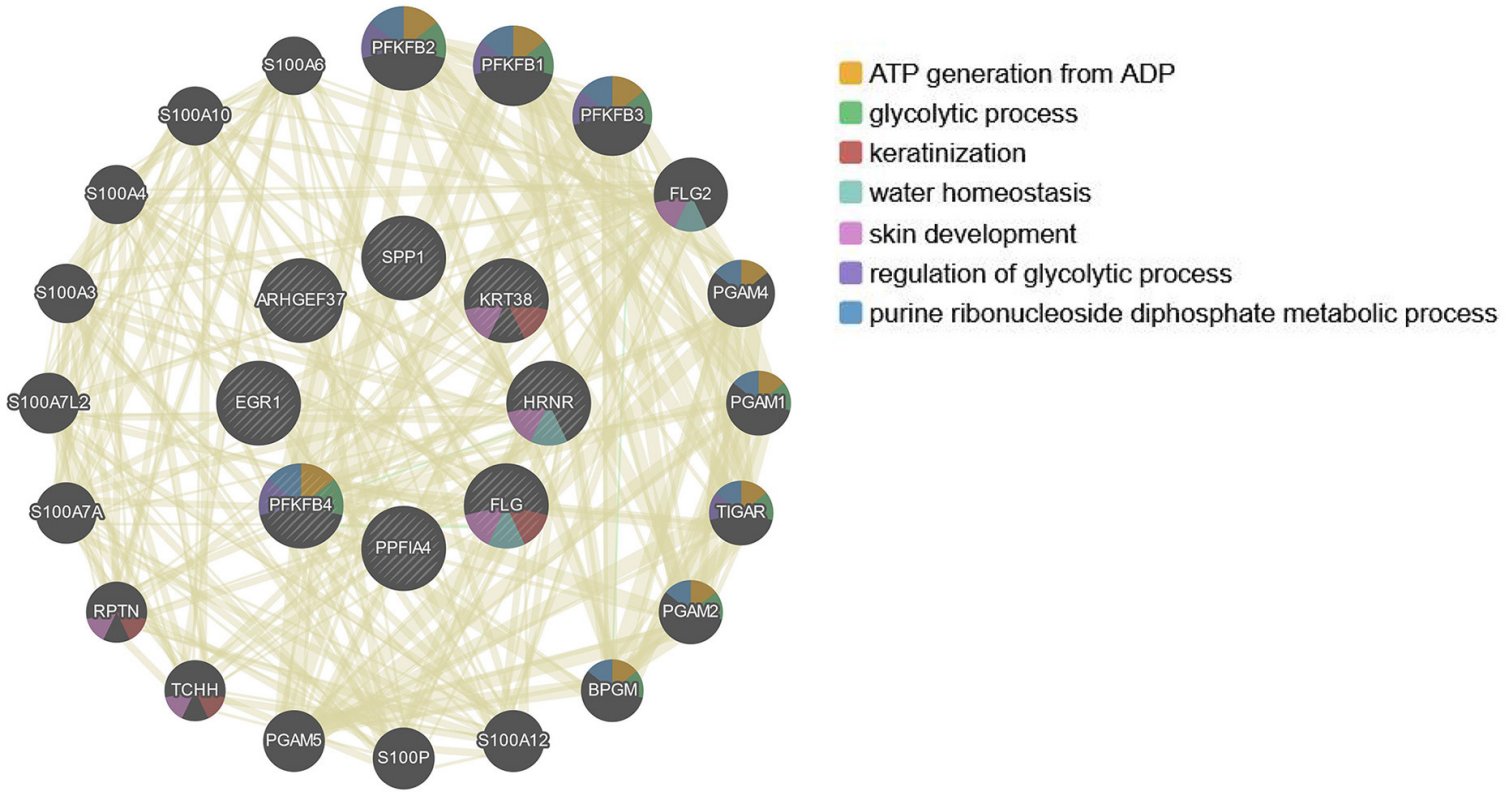
Relation network of the twelve hub genes analyzed by GeneMANIA.

**Figure 6:**
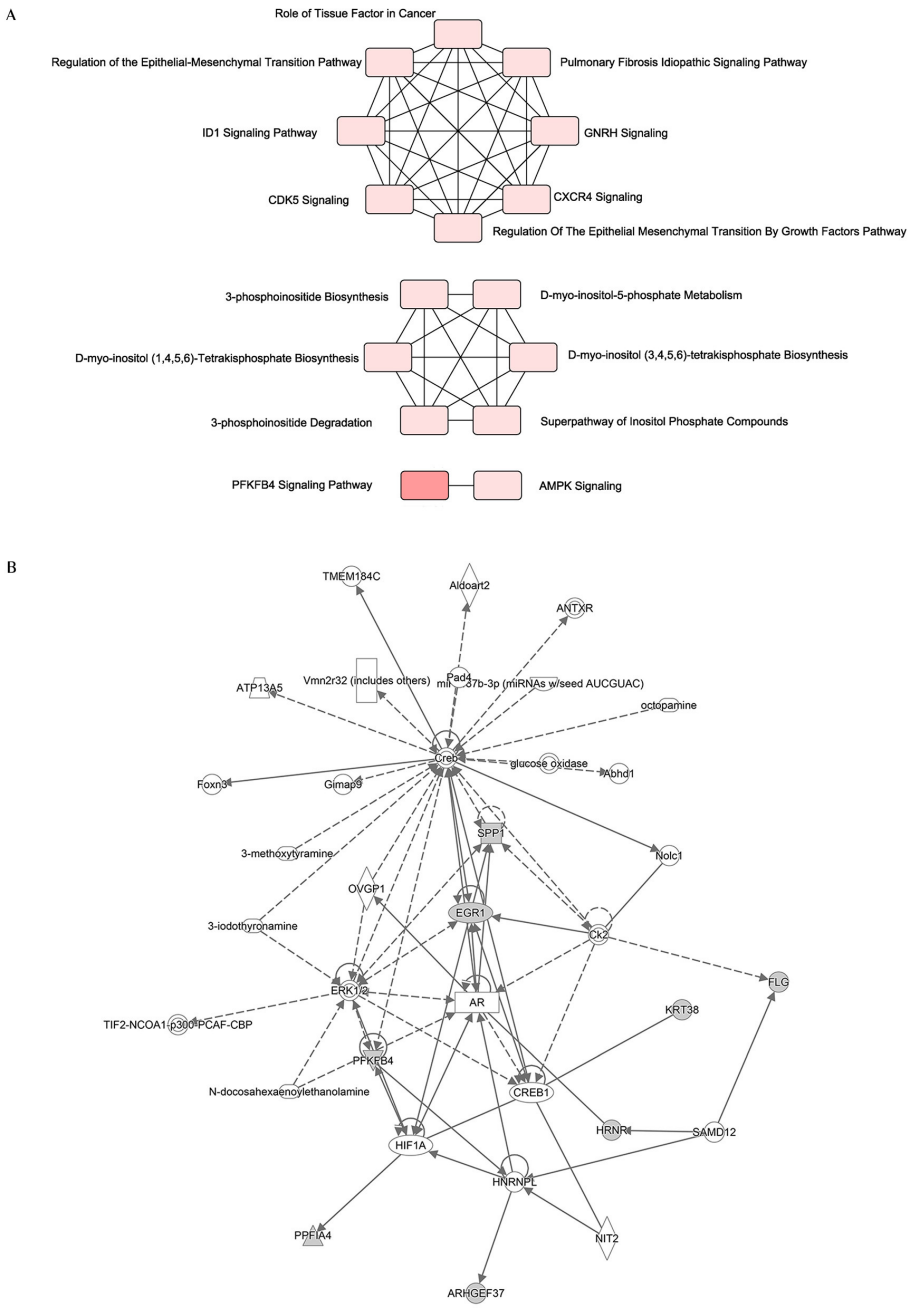
Functional network of the twelve hub genes. **(A)** An analysis of the core functions of twelve hub genes by QIAGEN Ingenuity Pathway Analysis (IPA). **(B)** An analysis of the core interaction network of twelve hub genes by IPA.

Since the 12 hub genes serve as potential targets of fullerenol treatment for aged-related macular degeneration (AMD) to investigate the potential roles and related genes of those genes, we performed GeneMANIA analysis and constructed a gene interaction network (Figure 5). In the outer circle, GeneMANIA identified functionally associated genes with eight of 12 hub genes (in the inner circle: *ARHGEF37, KRT38, FLG, HRNR, PPFIA4, PFKFB4*, and *SPP1*). GeneMANIA pathway analysis using the eight hub genes (the inner circle) connected to 20 associated genes (the outer circle) by genetic, physical, or pathway analysis identifies seven significant pathways (Figure 5). The top two pathways are ATP generation and glycolytic processes (Figure 5). Furthermore, ingenuity pathway analysis (IPA) showed detailed related biological pathways of the 12 crucial genes which include three major networks (Figure 6A). The first described a network characterizing signaling pathways including CXCR4 which is reported with the AMD pathogenesis (29, 30); the second included 3-phosphoinositide biosynthesis which is important for the initiation of early pathological events in retinal degenerative diseases under the presence of oxidative stress (31–33); the third included AMPK signaling which prevents degeneration of photoreceptors and the RPE cells (34, 35). In addition to a number of pathways, genes including *CREB, AR, ERK1*/2, *CREB1, HIF1A*, and *HNRNPL* genes, were also related to the 12 hub genes (Figure 6B). Among these genes, activation of *ERK1*/2 and *CREB* showed protection function for human RPEs from H_2_O_2_-induced oxidative damage (36). *AR* was proved to be a hub gene during the pathogenesis of AMD (37). These results provide further insights into the biological processes and potential roles regulated by the 12 hub genes. These targets and pathways may serve as potential targets for fullerenol treatment for AMD.

## Discussion

In this study, we first profiled the comprehensive gene expression levels of H_2_O_2_-induced senescent ARPE-19 cells by RNA sequencing. ROS were found to affect the expression of multiple genes, such as *CXCL8, SOD2, PLAT, CLSTN2, TXNIP, BIRC3, CLDN1*, and *DIO2*. We further demonstrated that in the presence of oxidants, fullerenol inhibited the steroid hormone-mediated signaling pathway, cell proliferation, cell migration, and negative regulation of gene expression pathways. On the other hand, genes influenced by fullerenol were involved in the positive regulation of DNA-templated transcription and the negative regulation of apoptotic process pathways to protect RPE senescence.

To explore the biological role of these affected genes between the H_2_O_2_-treated and fullerenol-treated groups, we annotated the GO functions of these genes. In our study, we found that genes involved in the negative regulation of apoptotic processes were down-regulated after H_2_O_2_ treatment and up-regulated after fullerenol treatment, indicating that the apoptotic process is activated by H_2_O_2_ damage but inhibited by fullerenol. A previous study supported that the apoptotic process of RPEs was changed after various damages, such as light irradiation and the progression of AMD. As described in our study, genes involved in the positive regulation of the DNA-templated transcription pathway were also down-regulated after H_2_O_2_ treatment and up-regulated after fullerenol treatment. This pathway may also play crucial roles in RPE cells after H_2_O_2_ and fullerenol treatment. For the first time, our results showed that genes in the steroid hormone mediated signaling pathway were up-regulated after H_2_O_2_ treatment and down-regulated after fullerenol treatment, indicating the effects of fullerenol on the steroid hormone signaling pathway in RPE cells. A previous study showed that the level of serum cortisol (a steroid hormone) was positively correlated with RPE alterations in diabetic retinopathy. In addition, there were three more pathways, whose genes were up-regulated after H_2_O_2_ treatment and down-regulated after fullerenol treatment: negative regulation of gene expression, cell proliferation, and cell migration pathways. These pathways play crucial roles in RPE cells after H_2_O_2_ and fullerenol treatment. Our study provides global insight into the transcriptome changes in RPE cells, and hub genes in those pathways have the potential to serve as targets of fullerenol treatment.

Notably, we identified nine genes (*ARHGEF37, KRT38, AC005307.1, FLG, HRNR, AC79466.1, PPFIA4, PFKFB4*, and *SPP1*) that were upregulated after H_2_O_2_ treatment and downregulated after fullerenol treatment, and three genes (*EGR1, AC010343.1, PFN1P1*) were downregulated after H_2_O_2_ treatment and upregulated after fullerenol treatment. The 12 genes play crucial roles in metabolism-related pathways and biological signaling pathways. For example, *ARHGEF37* was reported to serve as a regulatory protein involved in endocytosis (38). *SPP1* is related to the activation of the PI3K/AKT and ERK1/2 pathways (39). Our study revealed their new roles in the regulation of H_2_O_2_ damage and fullerenol treatment of RPE cells. They may serve as useful biomarkers to illustrate the potential functions of fullerenol.

This study has several limitations. Firstly, when compared with the control group, the fullerenol treatment group had more DEGs than the H_2_O_2_-induced group. We speculate that this is due to the function of fullerenol itself. A previous study used RNA sequencing analysis to confirm that fullerenol itself can alleviate corneal oxidative injury by downregulation of oxidative stress-associated genes and upregulation of proliferation-related genes (19). However, the function of fullerenol in RPE cells needs further investigation in the future. Secondly, we only did the relation network analysis of the 12 hub genes by GeneMANIA and IPA analysis. Therefore, further molecular and cellular studies are needed to confirm the mechanistic basis for our conclusions.

**Figure 7:**
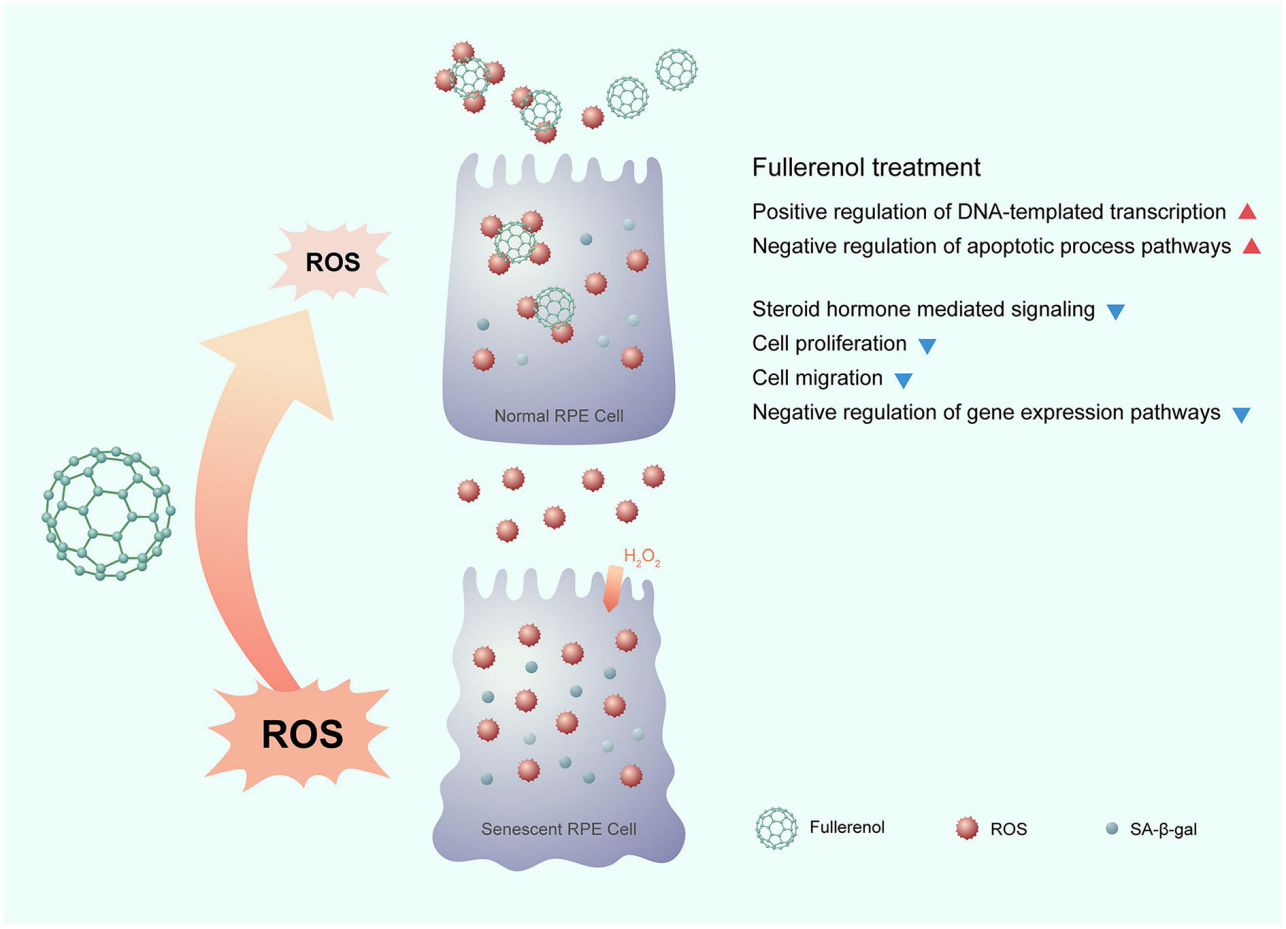
Potential mechanism of the nanomaterial fullerenol as an antioxidant in senescent RPE cell treatment.

In this study, we comprehensively profiled the gene expression of an H_2_O_2_-induced ARPE-19 senescence model and a nanoantioxidant fullerenol rescue model for the first time. Through dynamic transcriptome analysis, we identified positive regulation of the DNA-templated transcription and the negative regulation of apoptotic processes were down-regulated after H_2_O_2_ treatment and up-regulated after fullerenol treatment. In addition, there were four more pathways, whose genes were up-regulated after H_2_O_2_ treatment and down-regulated after fullerenol treatment: the steroid hormone mediated, negative regulation of gene expression, cell proliferation, and cell migration pathways. We also identified 12 hub genes that were rescued by fullerenol treatment. The 12 hub genes showed promise as therapeutic targets for AMD, which is worth further investigation. In summary, our results provide evidence for the nanomaterial fullerenol as an antioxidant in AMD treatment (Figure 7).

## Data availability statement

The raw data were submitted to the NCBI-SRA database with the Bioproject ID: PRJNA722601 (https://www.ncbi.nlm.nih.gov/bioproject/PRJNA722601).

## Author contributions

LM and QZ initiated the study. XW, FY, J-YX, and YL performed the data analysis. JC and WL performed cell culture. XW and FY wrote and revised the manuscript. JD discussed and optimized the pictures in this manuscript. ZP, LM, and QZ designed the study and revised the manuscript. All authors contributed to the article and approved the submitted version.

## Funding

This study was supported by Shenzhen High-level Hospital Construction Fund (2019).

## Conflict of interest

The authors declare that the research was conducted in the absence of any commercial or financial relationships that could be construed as a potential conflict of interest.

## Publisher's note

All claims expressed in this article are solely those of the authors and do not necessarily represent those of their affiliated organizations, or those of the publisher, the editors and the reviewers. Any product that may be evaluated in this article, or claim that may be made by its manufacturer, is not guaranteed or endorsed by the publisher.

## Supplementary material

Supplementary Figure S1 Gene expression distribution across all the samples. **(A)** Boxplot of the distribution of FPKMs of all the genes in each sample. **(B)** Density plot of FPKMs of all the genes in each group. **(C)** PCA plot for each sample based on FPKMs of all the genes. **(D)** Correlation heatmap plot of each sample based on FPKMs of all the genes. S, H_2_O_2_ group; C, Ctrl group; SF, Fullerenol group.

## Abbreviations

AMD: aged-related macular degeneration
RPE: retinal pigment epithelium
DEGs: differentially expressed genes
GSEA: gene set enrichment analysis
GO: Gene Ontology.
