# Supplementary figures and images for: The transcriptome profile of RPE cells by the fullerenol against hydrogen peroxide stress
Source: Front Med (Lausanne). 2022 Sep 14;9:996280. doi: 10.3389/fmed.2022.996280 (PMC9515647; doi:10.3389/fmed.2022.996280)

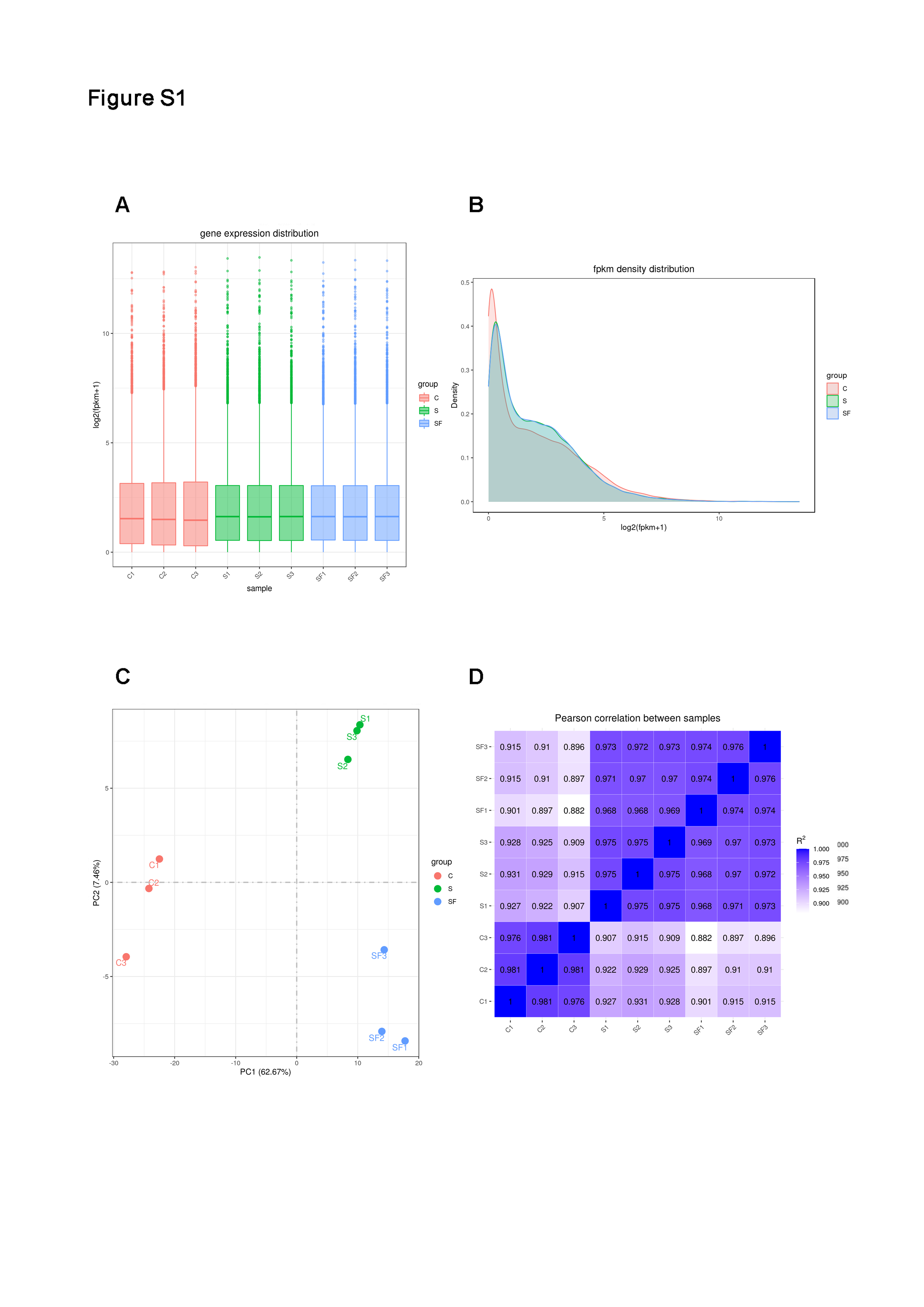

Supplement: Supplementary Figure S1 — Gene expression distribution across all the samples. (A) Boxplot of the distribution of FPKMs of all the genes in each sample. (B) Density plot of FPKMs of all the genes in each group. (C) PCA plot for each sample based on FPKMs of all the genes. (D) Correlation heatmap plot of each sample based on FPKMs of all the genes. S, H2O2 group; C, Ctrl group; SF, Fullerenol group. [file Image_1.TIFF]
